# Identification of *MPL* R102P Mutation in Hereditary Thrombocytosis

**DOI:** 10.3389/fendo.2017.00235

**Published:** 2017-09-20

**Authors:** Christine Bellanné-Chantelot, Matthieu Mosca, Caroline Marty, Rémi Favier, William Vainchenker, Isabelle Plo

**Affiliations:** ^1^INSERM UMR1170, Gustave Roussy, Villejuif, France; ^2^Department of Genetics, Assistance Publique-Hôpitaux de Paris (AP-HP) Hôpitaux Universitaires Pitié Salpêtrière—Charles Foix, UPMC Univ Paris 06, Paris, France; ^3^Université Paris-Saclay, UMR1170, Gustave Roussy, Villejuif, France; ^4^Gustave Roussy, UMR1170, Villejuif, France; ^5^Assistance Publique-Hôpitaux de Paris (AP-HP), Service d’Hématologie biologique, Centre de Référence des Pathologies Plaquettaires (CRPP), Hôpital Armand Trousseau, Paris, France

**Keywords:** thrombopoietin, JAK2, MPL R102P, thrombocytosis, thrombocytopenia, CAMT

## Abstract

The molecular basis of hereditary thrombocytosis is germline mutations affecting the thrombopoietin (TPO)/TPO receptor (MPL)/JAK2 signaling axis. Here, we report one family presenting two cases with a mild thrombocytosis. By sequencing *JAK2* and *MPL* coding exons, we identified a germline *MPL* R102P heterozygous mutation in the proband and his daughter. Concomitantly, we detected high TPO levels in the serum of these two patients. The mutation was not found in three other unaffected cases from the family except in another proband’s daughter who did not present thrombocytosis but had a high TPO level. The *MPL* R102P mutation was first described in congenital amegakaryocytic thrombocytopenia in a homozygous state with a loss-of-function activity. It was previously shown that MPL R102P was blocked in the endoplasmic reticulum without being able to translocate to the plasma membrane. Thus, this case report identifies for the first time that *MPL* R102P mutation can differently impact megakaryopoiesis: thrombocytosis or thrombocytopenia depending on the presence of the heterozygous or homozygous state, respectively. The paradoxical effect associated with heterozygous *MPL* R102P may be due to subnormal cell-surface expression of wild-type MPL in platelets inducing a defective TPO clearance. As a consequence, increased TPO levels may activate megakaryocyte progenitors that express a lower, but still sufficient level of MPL for the induction of proliferation.

## Background

Hereditary thrombocytosis is characterized by an increase in platelets above 450 × 10^9^/L due to germline genetic abnormalities. These mutations affect the thrombopoietin (TPO)/TPO receptor (MPL)/JAK2 axis that is essential for megakaryopoiesis. First, heterozygous mutations in the *THPO* gene located in the 5′-untranslated region or in splice donor sites lead to an increased mRNA translation and synthesis of TPO (1–3). Second, several heterozygous *JAK2* mutations were discovered that are located not only in the pseudokinase but also in the kinase domains such as *JAK2* H608N, *JAK2* R564Q, *JAK2* S755R, *JAK2* R938Q, and *JAK2* R867Q (4–7). These mutants harbor low constitutive kinase activity that is dependent on the presence of MPL (5). Third, *MPL* mutations were identified affecting different residues of the receptor and involving different mechanisms. The heterozygous gain-of-function mutation *MPL* S505N was identified in the transmembrane domain of MPL and was found to induce its dimerization and activation (8). Interestingly, loss-of-function mutations have also been identified including the homozygous *MPL* P106L in Saoudian and Koweitian families (9, 10). Studies have shown that MPL P106L presents a defective trafficking to the cell membrane leading to a decrease in TPO clearance by platelets, which results in high circulating TPO levels that induce a proliferative response to TPO in immature cells (9, 10). The *MPL* K39N polymorphism was also discovered in 7% of African Americans and homozygous cases present a thrombocytosis associated with an abnormal MPL maturation (11).

## Case Presentation

We have identified two members of a family displaying mild thrombocytosis diagnosed at an early age (21 and 41 years old) with platelet counts around 600 × 10^9^/L. Four other members of the family were included in the study and the pedigree was registered in the National collection of myeloproliferative neoplasms (MPNs) (DC 2009-957). All participants gave their written informed consent in accordance to the Declaration of Helsinki and the study approved by Ethical committee of Ile de France (no. IDRCB 2008-A00658-47). Clinical and biological annotations were recorded in an Access database approved by the French computer commission (CNIL #815419).

**Figure 1:**
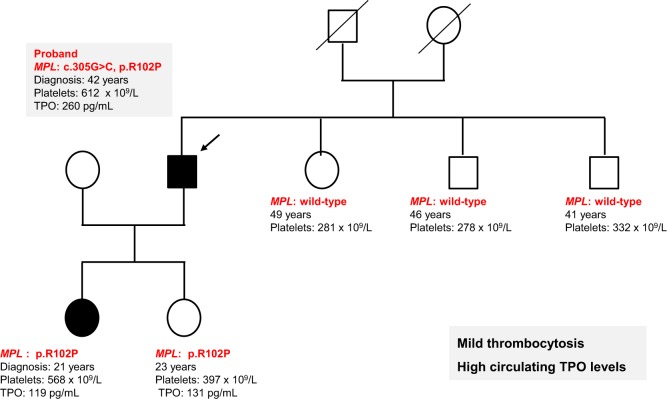
Heterozygous *MPL* R102P mutation induces a mild thrombocytosis with high thrombopoietin (TPO) levels. Pedigree from a family harboring a mild thrombocytosis with platelet counts around 600 × 10^9^/L. The proband indicated by an arrow was diagnosed at 42 years old and one of her daughter was 21 years old. *MPL* was sequenced and a heterozygous *MPL* R102P mutation was identified. TPO levels were found elevated 119 and 260 pg/mL. Another daughter presented a high TPO level 131 pg/mL but without disease.

All exons of *MPL* and *JAK2* genes were sequenced in the proband’s DNA extracted from total blood cells (indicated by an arrow in Figure 1) using the BigDye Terminator chemistry (Life Technologies) on an ABI3730 Genetic Analyser (Applied Biosystem). We found a heterozygous *MPL* R102P mutation that had been previously reported in congenital amegakaryocytic thrombocytopenia (CAMT) but was previously found to be in a homozygous or compound heterozygous state leading to a complete defect in MPL function (12–14). This *MPL* R102P mutation was also identified in the other affected case demonstrating that this event was germline. TPO levels were measured by ELISA in the two affected subjects and were found elevated (119 and 260 pg/mL) compared to the normal range (<30 pg/mL). The mutation was not found in the other non-affected members of the family except in the second 23-year-old proband’s daughter. This case had a normal but rather high level of 397 × 10^9^/L platelets and a high TPO level of 131 pg/mL. However, this asymptomatic carrier has not reached the father’s age at diagnosis and thus will be carefully followed in clinics. These results suggest an incomplete penetrance of the *MPL* mutation depending on age.

## Discussion

MPL is a homodimeric type 1 receptor that controls megakaryopoiesis after TPO binding. It is expressed at high levels in hematopoietic stem cells, megakaryocytic (MK) progenitors, megakaryocytes, and platelets, while the TPO levels are controlled in large part by the platelet mass due to internalization of TPO/MPL/JAK2 complexes.

Many mutations affecting *MPL* induce deregulation of megakaryopoiesis. Although some are gain-of-function, such as *MPL* W515K/L and S505N, leading to MPNs or hereditary thrombocytosis (8, 15), others such as *MPL* R102P and F104S are loss-of-function leading to CAMT (12–14). The MPL R102P receptor function was previously investigated and studies have shown that it is not addressed to the cell-surface and induces no proliferation of Ba/F3 cell lines (14). This loss of function was caused by the presence of an abnormal glycosylated receptor resulting from its blockade in the endoplasmic reticulum (ER) (12–14). In CAMT, *MPL* mutations are recessive events (homozygous or compound heterozygous state) generally located throughout the *MPL* gene, implying that no functional MPL receptors are present at the cell surface.

**Figure 2:**
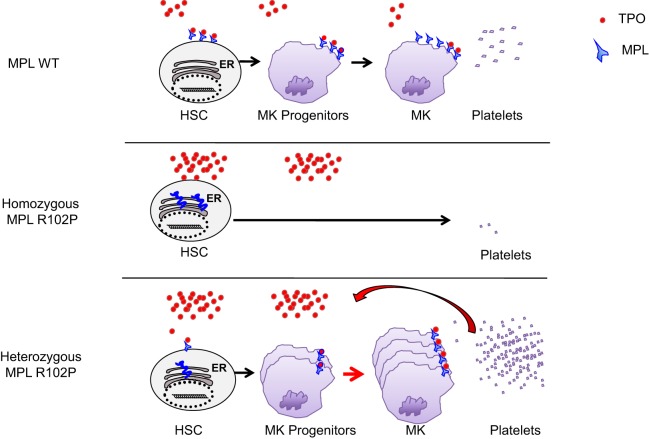
Model of MPL R102P function. In wild-type, MPL is increasingly upregulated in megakaryocytic progenitors (MK progenitors) and platelets. The level of thrombopoietin (TPO) is controlled by the platelet mass due to the internalization and degradation of TPO/MPL/JAK2 complexes. In homozygous *MPL* R102P situation, MPL cannot signal to induce megakaryopoiesis and platelets production even if the TPO level is high since it is blocked in the endoplasmic reticulum (ER). In *MPL* R102P heterozygous condition, only wild-type MPL is expressed at the cell surface leading to subnormal expression in platelets and defective TPO clearance. Therefore, the TPO levels increased and TPO is able to favor the proliferation of MK progenitors through the wild-type MPL, leading to a thrombocytosis.

This case report identifies for the first time that *MPL* R102P mutation can differently impact megakaryopoiesis depending on heterozygous and homozygous states (Figure 2). Indeed, based on previous studies showing that MPL R102P remains in the ER, we propose that in the heterozygous state, only the wild-type MPL form is addressed to the cell surface leading to subnormal expression in platelets and a consequent deficit in TPO clearance. Therefore, heterozygous *MPL* R102P results in an increased TPO levels in the serum, which is able to induce the proliferation of MK progenitors expressing sufficient MPL. Altogether this phenomenon leads to thrombocytosis by a decreased TPO clearance, as has also been demonstrated in transgenic mice with a low expression of MPL in megakaryocytes and platelets (16, 17).

## Concluding Remarks

We identified one family of hereditary thrombocytosis with three members harboring a germline *MPL* R102P heterozygous mutation with high TPO levels in the serum but with incomplete penetrance of the phenotype. These results contrast with the presence of the same *MPL* R102P mutation at homozygous state in CAMT.

## Ethics Statement

Four other members of the family were included in the study, and the pedigree was registered in the National collection of myeloproliferative neoplasms (MPNs) (DC 2009-957). All participants gave their written informed consent in accordance to the Declaration of Helsinki and the study approved by Ethical committee of Ile de France (No. IDRCB 2008-A00658-47). Clinical and biological annotations were recorded in an Access database approved by the French computer commission (CNIL #815419).

## Author Contributions

C-BC, IP, and WV designed the work and wrote the manuscript. MM and RF performed experiments. CM performed experiments and contributed to the writing of the manuscript.

## Conflict of Interest Statement

The authors declare that the research was conducted in the absence of any commercial or financial relationships that could be construed as a potential conflict of interest.
